# Ablation of the Jugular Vein

**Published:** 1902-10-04

**Authors:** 


### Hospital Clinics and Medical Progress.

### ABLATION OF THE JUGULAR VEIN.

In a very striking paper Mr. Charles Balance,[1] of St. Thomas's Hospital, shows that occlusion of the lateral sinus and internal jugular vein is an essential part of the method employed by nature, and to be employed by the surgeon in imitation of nature, for arrest of acute general infection having its origin within the temporal bone. There can be no question that there is a wide divergence of opinion on the subject of tying the vein. Speaking of what he saw in a recent tour, he says that in one clinic it was tied, in another it was never tied, in a third it was not dealt with unless symptoms persisted after the temporal bone operation had been carried out, while in yet a fourth it was strongly urged that the lateral sinus and internal jugular vein should be slit open for the whole extent occupied by infected clot, even should this involve an operation extending from the torcular to the subclavian. No one again will contradict the statement that in Great Britain a like divergence of opinion prevails. All true and lasting surgical practice, however, is based upon pathology, and when once the pathology of an affection is clearly appreciated, divergence of view as to treatment ought to vanish. The principle upon which the operation upon the vein is based—namely the occlusion of the main venous channel leading from an infected area—is not simply one of local applicability; indeed, when we turn to the pathology of the disease we find that the sinus thrombosis itself is really a protective measure, and that when the clot remains uninfected it is successful. The thrombus may however become infected, and the disease will then

"Break out into a second course of mischief."

Thus is set up an additional source of danger, often of such gravity that it overshadows the primary disease. It is the secondary infection of the clot and not the clot itself which constitutes the danger in such cases.

Nature's process for the prevention of general infection is sequestration of the infective material, as is shown by the formation of local abscess. Thus nature surrounds the poisoned tissues, be they in bone, in dura, or in blood (by coagulation), with an inflammatory barrier; and the surgeon thus learns how surgical art may deal successfully with acute septicæmia and acute pyæmia. Nor should we in such cases endeavour to differentiate too closely between the cases; for if after the operation on the temporal bone the signs of general infection should persist, an error of judgment which cannot then be rectified has been committed in not completing the operation. It is true that the vein may now be tied, and that some such cases have been successful:

"Fortune brings in some boats that are not steer'd."

But less can be expected from the operation on the vein when done as a secondary measure than when carried out as a part of the first operation. Greig Smith said in reference to the operation for the relief of intestinal obstruction: "Let it be either drugs or operation, and never that fatal compromise—operation when drugs fail." So here let the surgeon make unflinching decision whether the case be one calling for operation on the vein as well as on the temporal bone, and not adopt that fatal compromise—operation on the vein when operation on the temporal bone has proved insufficient.

Operation on the vein should then be undertaken (*a*) in acute pyæmia and acute septicæmia, whether the sinus is occupied by clot or by fluid blood; (*b*) if the sinus wall is gangrenous or its contents are putrefying, unless it is perfectly clear that on both sides of the area of inflammation the lumen of the sinus is completely blocked by non-infected thrombi (how seldom this qualification will be operative will be obvious to those who have most experience of these cases); (*c*) if it is proved, or even suspected, that the blood of the jugular bulb is in part or wholly clotted; and (*d*) if the jugular vein is thrombosed. When, before beginning to operate, it has been decided to deal with the vein, the operation on the vein should precede that on the temporal bone, and when operation on the vein is first decided upon during the course of the temporal bone operation, it should be forthwith carried through without waiting for the completion of the operation on the sinus region.

The vein, when not thrombosed, is exposed in the neck about the level of the cricoid cartilage, and the surgeon, tying its tributaries and dividing them between two ligatures, should remove it completely up to the bulb. Ablation of the vein is better than ligatures, but it involves ligature of the tributary veins; and as the line of incision involves the external jugular, that vessel is also necessarily tied. In cases of thrombosis of the internal jugular vein its ablation begins at its junction with the subclavian, and it will not be difficult to find the vertebral vein on its inner side, lying on the scalenus anticus, and to tie and divide it also. As for the sinus, it is impossible to deal with this as one does with the vein—that is by removal—as this would expose the intradural space. The obvious proceeding is to slit up the sinus, if necessary from torcular to bulb, so that the whole area of infective inflammation may be exposed and dressed.

[1] Lancet, Sept. 20.

